# Chemistry of the Four Seasons: Spring, Summer Autumn, Winter; an Essay Principally Concerning Natural Phenomena Admitting of Interpretation by Chemical Science, and Illustrating Passages of Scripture

**Published:** 1846-12

**Authors:** 


Art. VI.—*Chemistry of the Four Seasons: Spring, Summer, Autumn, Winter; an Essay principally concerning natural phenomena admitting of interpretation by Chemical Science, and illustrating passages of Scripture.* By Thomas Griffiths, Professor of Chemistry in the Medical College of St. Bartholomew's Hospital; Author of "Recreations in Chemistry," and "Chemistry of the Four Elements." Philadelphia: Lea & Blanchard. 1846. 12mo. pp. 451.

They greatly err who suppose that Chemistry is confined to the Laboratory. It is no technical science, limited to processes of art—to the preparation of medicines, the extraction of metals, to bleaching, dying, the manufacture of glass, the analysis and amelioration of soils—but a science wide in its scope and nature. It takes in all that passes in the four seasons, in air, earth, and water. In spring time the seeds are sown, and chemical changes, under the transforming influence of caloric, oxygen, and water, commence. The starch which they contain is converted into sugar and mucilage, their oil into carbonic acid; as they grow, carbonic acid again takes the shape of oil and sugar, and as their seeds ripen, the sugar returns to starch again. In their fruits changes occur by which bitterness and astringency give place to the saccharine principle. The *acer saccharinum* is tapped in the spring and yields a sap from which sugar is extracted. After a time the saccharine matter declines and sugar can no longer be crystalized from the juice of the tree; but molasses, or uncrystallizable sugar, may still be obtained. Finally the sweet principle is lost, and the tree exudes a sap abounding in mucilage. After a time this is transformed into leaves and woody fibre, and the tree is clothed in green.

Has the reader ever visited a great cave—the Mammoth, for example? If so, he has seen not only a vast labyrinth, but an extended laboratory, itself worked out by chemical action. There, in one part, he remarked water wearing away the solid rock, and in another depositing the limestone in stalactites of every strange, fantastic shape. At another point he found sulphate of lime, as snowy gypsum, covering the ceiling with brilliant incrustations. Again, he saw sulphate of soda deposited upon the walls and floor; and close by found the crystals of sulphate of magnesia. From one fountain he drank water impregnated with sulphate of magneisum, and from another water containing sulphate of iron. By chemical agency the cave itself has been scooped out of the compact limestone rock, and this silent power is ceaselessly at work in its dark chambers, effecting new combinations, dissolving stone, creating new salts, crystallizing what was amorphous, rendering active what was inert.

These are examples. The book before us is full of such. It illustrates on every page the truth of our remark, that chemistry is no narrow science, confined to the laboratory and workshop. It is a book which will be read with pleasure, and is especially suited to the general reader. It does not profess to be competent to teach the adept in chemical science.

The following extract will convey an impression as to the subjects and style of this work, and will lead some, we should hope, to avail themselves of this pleasant guide to the study of the chemistry of nature.

"Upon tracing lignin as a chief constituent of the bark of trees, the chemist discovers another example of the power and goodness of the Creator.

"Lignin is an exceedingly bad conductor, and therefore only very slowly permeable by heat or cold; thus the covering of bark prevents the sap of trees from disturbance in its circulation by sudden changes in the temperature of the air, and secures it the degree most congenial to the performance of its vital functions.

"If the bark were not thus created of bad conducting material, trees and plants would suffer from the heat of summer, and the cold of winter; but more especially from the latter, as it would cause their death by the freezing of the sap, a phenomenon sometimes observed during a protracted and rigorous winter; but it can be prevented in many instances by enveloping the trunks of trees with a thicker coating of woody fibre, in the form of matting, or bands of hay and straw.

"When tropical plants are transported to cold climates, we artificially clothe them with similar materials to prevent them from suffering by the sudden transition, the same as we should endeavor to protect ourselves, by putting on an extra garment upon passing from hot to cold weather.

"By keeping a thermometer plunged in a hole bored in a sound tree during hot weather, it constantly indicated a temperature several degrees below that of the atmosphere; and during cold weather a temperature several degrees above that of the atmosphere: and another wonderful fact was also discovered, namely, that sap drawn from the tree would freeze at the same temperature as pure water, whilst the sap circulating in the tree, under vital agency, would not freeze until reduced seventeen degrees below the freezing point of water. This point or degree will claim our attention when considering the leading chemical phenomena of winter.

"It is a generally received and probably correct statement, that heat is evolved during germination,—heat quite independent of that which is popularly known to be evolved during putrefactive fermentation, as that of damp hay, damp corn, or organic manures;—the temperature of plants is very frequently materially above that of the atmosphere, 'and all have undoubtedly a power dependent upon vitality of maintaining to a great extent an equable temperature.'

"In summer and autumn the dense foliage of trees, by its extraordinary non-conducting power, forms the cool shade which at noon-day is so eagerly sought by man and animals from the piercing rays of the sun, and more particularly in oriental climates.

"The chemist discovers that the leaves of some very succulent plants will absorb oxygen in the dark, and again evolve it in the light; some kinds of the 'cactus,' for example, will absorb more than their own bulk of oxygen, and retain it so tenaciously, that they will not yield it by the action of heat, nor in the vacuum of an air-pump, but immediately do so upon exposure to the imponderable agency of solar light.

"No satisfactory evidence can be adduced regarding the physical cause of the circulation of the sap in plants, nor even of the general direction of its course; but the most accurate researches which have been made upon this recondite subject, render it probable that the chief ascent of the sap from the roots to the leaves, is in the vascular system of the 'alburnum,' and outer layers of wood; and that having performed its functions in the leaves, as shown at page 84, it chiefly descends by the 'liber,' or inner layer of bark, giving rise to the formation of new wood, and to the separation of gum, resin, and other proximate principles.

"As regards the influence of flowers upon the atmosphere, it appears that they deteriorate it to some extent, and particularly during the night; but it is highly probable that much of the oppression which persons frequently experience after remaining in a conservatory, or room filled with flowers, is in the first place referable to the confined atmosphere being loaded with carbonic acid arising from the tan and soil; and in the next from the concentrated aroma of the flowers, and not from any material deterioration of air by their agency.

"Some persons are more affected by the aroma of flowers than others; thus a single hyacinth in a large room certainly cannot deteriorate the air in any appreciable degree, and yet its odor may be extremely oppressive, producing even headache and faintness.

"Few persons can bear an atmosphere loaded with aroma, whilst a little is occasionally refreshing; the same as a pocket-handkerchief saturated with otto of rose, soon palls upon the senses, though the first diffusion of its odor might have been grateful."

